# Inferior Vena Cava Measurement with Ultrasound: What Is the Best View and Best Mode?

**DOI:** 10.5811/westjem.2016.12.32489

**Published:** 2017-02-24

**Authors:** Nathan M. Finnerty, Ashish R. Panchal, Creagh Boulger, Amar Vira, Jason J. Bischof, Christopher Amick, David P. Way, David P. Bahner

**Affiliations:** The Ohio State University College of Medicine, Department of Emergency Medicine, Columbus, Ohio

## Abstract

**Introduction:**

Intravascular volume status is an important clinical consideration in the management of the critically ill. Point-of-care ultrasonography (POCUS) has gained popularity as a non-invasive means of intravascular volume assessment via examination of the inferior vena cava (IVC). However, there are limited data comparing different acquisition techniques for IVC measurement by POCUS. The goal of this evaluation was to determine the reliability of three IVC acquisition techniques for volume assessment: sub-xiphoid transabdominal long axis (LA), transabdominal short axis (SA), and right lateral transabdominal coronal long axis (CLA) (aka “rescue view”).

**Methods:**

Volunteers were evaluated by three experienced emergency physician sonographers (EP). Gray scale (B-mode) and motion-mode (M-mode) diameters were measured and IVC collapsibility index (IVCCI) calculated for three anatomic views (LA, SA, CLA). For each IVC measurement, we calculated descriptive statistics, intra-class correlation coefficients (ICC), and two-way univariate analyses of variance.

**Results:**

EPs evaluated 39 volunteers, yielding 351 total US measurements. Measurements of the three views had similar means (LA 1.9 ± 0.4cm; SA 1.9 ± 0.4cm; CLA 2.0 ± 0.5cm). For B-Mode, LA had the highest ICC (0.86, 95% CI [0.76–0.92]) while CLA had the poorest ICC (0.74, 95% CI [0.56–0.85]). ICCs for all M-mode IVCCI were low. Significant interaction effects between anatomical view and EP were observed for B-mode and M-mode measurements. Post-hoc analyses revealed difficulty in consistent view acquisition between EPs.

**Conclusion:**

Inter-rater reliability of the IVC by EPs was highest for B-mode LA and poorest for all M-Mode IVC collapsibility indices (IVCCI). These results suggest that B-mode LA holds the most promise to deliver reliable measures of IVC diameter. Future studies may focus on validation in a clinical setting as well as comparison to a reference standard.

## INTRODUCTION

Intravenous fluid resuscitation is vital in the critically ill; 1 however, excessive fluid administration has been shown to contribute to mortality.^2^,^3^ Rapid assessment of volume status may reduce over-resuscitation and improve outcomes. As it has been established that clinical examination alone is unreliable, more objective means of intravascular volume assessment have arisen. 4–6 Of those, point-of-care ultrasound (POCUS) of the inferior vena cava (IVC) has gained popularity as a noninvasive, easily obtainable, and rapid means of intravascular volume assessment. 7–10 Various techniques for IVC assessment have emerged but vary in populations studied, anatomical approach, and sonographic methodology.^7^,^8^,^11^–^14^ Currently there is no standardized approach for intravascular volume assessment by POCUS of the IVC, which may contribute to current controversies regarding its usefulness.^15^,^16^ The objective of this study was to quantify the difference between three approaches to IVC diameter measurement.

## METHODS

### Design

This was a prospective evaluation of 39 healthy adults approved by the hospital’s institutional review board.

### Setting and Population

Medical students from The Ohio State University College of Medicine participating in the Trained Simulated Ultrasound Patients (TSUP) program were enrolled on a volunteer basis and consented for participation in this study. Participating medical students serve as trained simulated ultrasound patients and are a volunteer group that fulfills the need for normal anatomic models for ultrasound education. 17 Exclusion criteria included inability to lie flat and inability by the ultrasonographer to adequately visualize and measure the IVC.

**Population Health Research Capsule**

What do we already know about this issue?

Though point-of-care ultrasound has gained popularity as a non-invasive means of intravascular volume assessment, there is no standardized approach to inferior vena cava measurement.

What was the research question?

Which anatomical view and imaging modality of the inferior vena cava has the highest inter-rater reliability?

What was the major finding of the study?

The sub-xiphoid transabdominal long axis view in gray scale (B-mode) demonstrated the highest inter-rater reliability.

How does this improve population health?

A standardized approach to non-invasive volume assessment may reduce discrepancies and variability in the acute healthcare of various populations.

### Protocol

Three emergency physician (EP) sonographers, all with experience in IVC POCUS (>150 ultrasounds performed), performed the ultrasound examinations. Two of the EPs are Registered Diagnostic Medical Sonographer (RDMS)-certified, and the third EP was completing an emergency medicine fellowship in ultrasound. Measurements of the IVC were obtained with the patient in the supine position. Data collection consisted of gray scale (B-mode) and motion-mode (M-mode) IVC diameter. For M-mode, IVC diameters were measured both during quiet passive respiration and then followed by a rapid inspiratory effort or “sniff.” Respiratory variability with percentage collapse of the IVC was calculated as the inferior vena cava collapsibility index (IVCCI): [(Maximum IVC diameter – Minimum IVC diameter)/Maximum IVC diameter] × 100.

**Figure 1:**
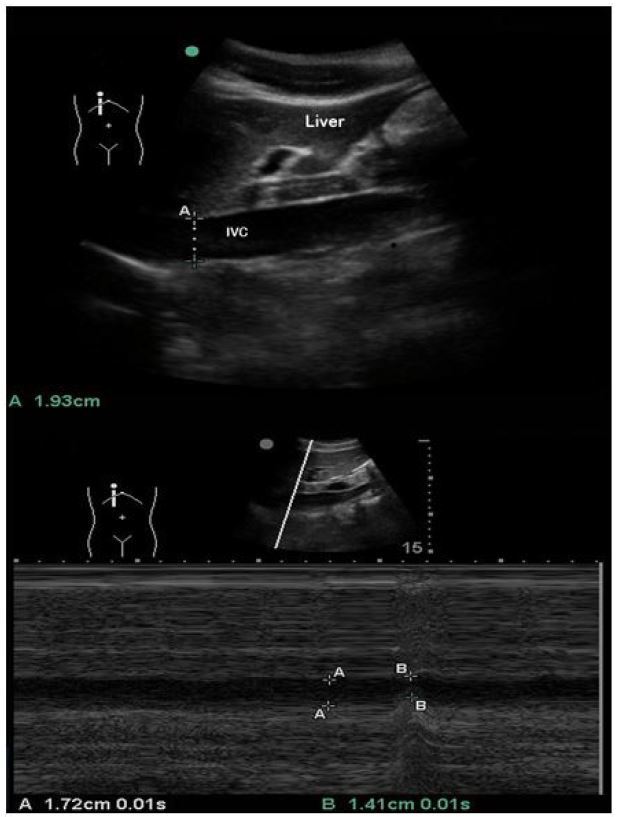
Sub-xyphoid transabdominal long axis (LA) in B-mode (top) and M-mode with respiratory variation (bottom). A: passive respiration, B: inspiratory effort. *IVC,* inferior vena cava

**Figure 2:**
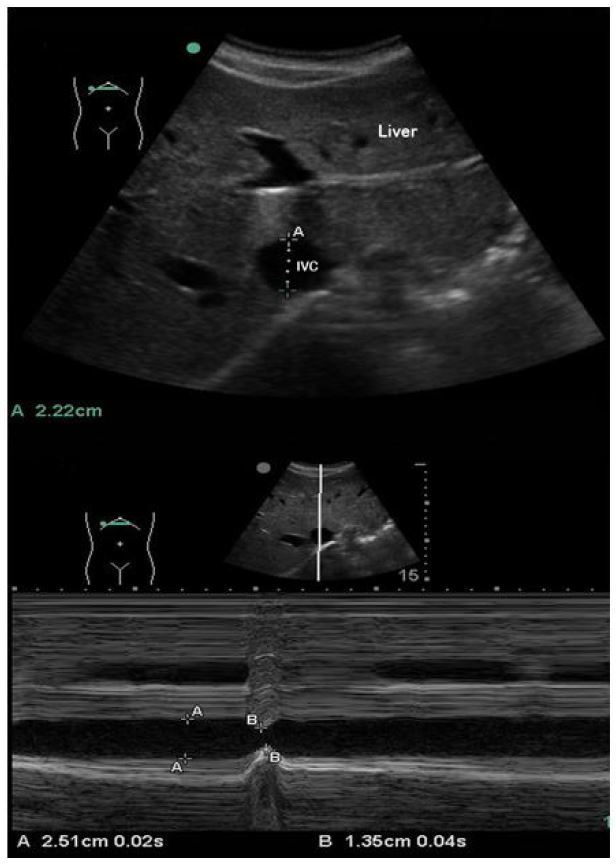
Transabdominal short axis (SA) in B-mode (top) and M-mode with respiratory variation (bottom). A: passive respiration, B: inspiratory effort. *IVC,* inferior vena cava

**Figure 3:**
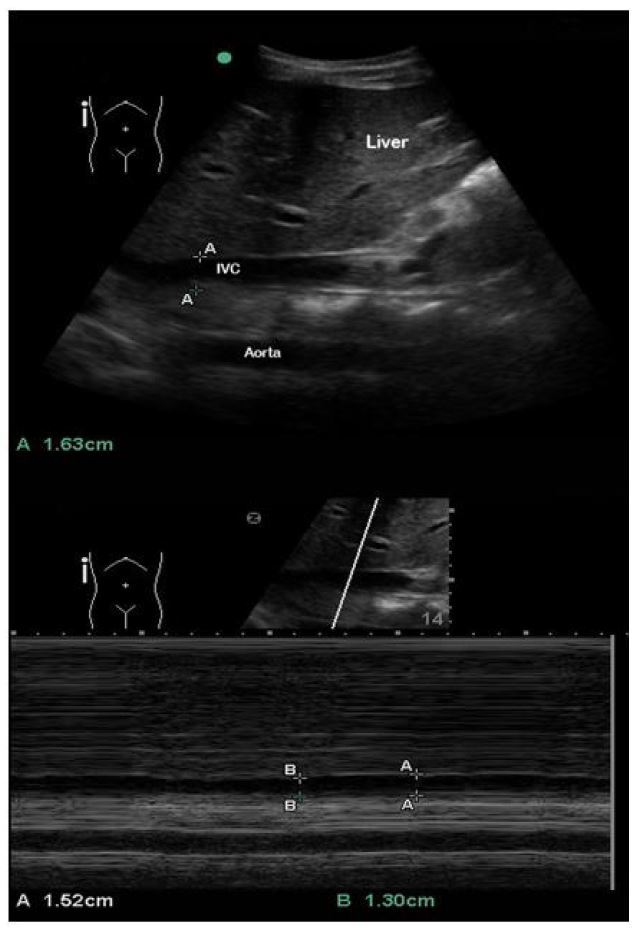
Right lateral transabdominal coronal long axis (CLA) (aka “rescue view”) in B-mode (top) and M-mode with respiratory variation (bottom). A: passive respiration, B: inspiratory effort. *IVC,* inferior vena cava

Three anatomic approaches were used for data collection and comparison: 1) sub-xyphoid transabdominal long axis (LA) 2–3cm caudal to the right atrial (RA) junction (Figure 1); 2) transabdominal short axis (SA) immediately inferior to the inflow of the hepatic veins (Figure 2); and 3) right lateral transabdominal coronal long axis (CLA) (aka “rescue view”) 2–3cm caudal to the RA junction (Figure 3).

All measurements were obtained with a 3.5-Mhz curved array ultrasound (US) probe on a portable US device (M-Turbo-Fujifilm – Bothell, Washington). Data were recorded in both digital and analogue formats and reviewed for quality assurance. For discrepancies in recorded data, we discarded analogue measurements and included digital data for analysis.

### Data Analysis

We calculated inter-rater reliability for each POCUS method using intra-class correlation coefficients (ICC) for continuous variables. In addition, the effects of sonographer and view acquisition on ICC values were analyzed via two-way univariate analyses of variance (ANOVA) with one repeated measure (EP by View) for both B- and M-mode to account for conditional changes imposed by the EP and method of acquisition. Significant main effects were followed up with post hoc analyses (Student Newman-Keuls (SNK)) and significant interactions were followed up with simple interactions. We performed statistical analysis using STATA v.12 (STATA Corp, College Station, TX). A sample size of 39 subjects was determined to have >80% power to detect a statistically significant difference in IVC measurement, with significance defined as alpha of 0.05.

## RESULTS

Each of the three EPs evaluated 39 TSUPs who were included in final statistical analysis, representing 351 total ultrasound scans. None of the volunteers met exclusion criteria. Mean diameters were performed for B-Mode, expiratory M-mode (IVCe), and inspiratory M-mode (IVCi) (Table 1). The highest ICC was found to be B-mode LA, 0.86 (95% confidence interval [CI] [0.76–0.92]) and poorest was M-mode IVCCI LA, 0.14 (95% CI [−0.27–0.47]) (Table 2).

We performed univariate ANOVA for each anatomic position and modality. Significant interaction effects between anatomical view and EP were observed for B-mode (p interaction < 0.01), IVCe (p interaction < 0.01), IVCi (p interaction < 0.01). Post hoc analyses revealed difficulty in consistent view acquisition between EPs.

## DISCUSSION

There are limited data comparing acquisition techniques. Wallace et. al. demonstrated equivalence in two anatomical approaches, namely, at the level of the left renal vein and 2 cm caudal to the hepatic vein inlet, both of which differ from measurements taken at the junction of the right atrium (RA). 7 The most commonly cited approaches are 2–3cm caudal to the RA junction and inferior, caudal, or distal to the hepatic veins, suggesting the need to compare these approaches.^14^,^18^–^26^

In this study we found strong agreement between EP sonographers for B-mode IVC diameter measurements and moderate agreement for IVCe and IVCi, measurements. Agreement between IVCCI was poor. Fields et. al. also described a strong agreement in IVC measurements when comparing diameter dimensions, which is subsequently lost in IVCCI analysis. This was ascribed to multiplicative augmentation in diameter differences in the IVCCI calculation leading to a lowering of ICC when comparing IVCCI to its separate elements. 27

Movement of the IVC occurs mediolaterally and craniocaudally during respirophasic POCUS, with collapse of the vessel occurring off axis from the true vertical. 28 This has led to suggestions in methodological approaches to IVC measurement favoring B-mode and discouraging M-mode^7^,^8^, although recent literature indicates that this may not be of clinical significance. 8 Our results do support the use of B-mode over M-mode; however, ICC remains moderate in IVCe and IVCi.

Our data suggest that B-mode, subxyphoid LA 2–3cm caudal to the RA junction is the most reliable means of IVC acquisition. When compared to SA immediately inferior to the hepatic veins and CLA (aka “rescue view”) 2–3cm caudal to the RA junction, LA has the highest ICC. IVC measurement is less reliable in M-mode when compared to B-mode. This discrepancy is augmented when calculating IVCCI. These findings are consistent with current literature on the topic.^8^,^14^,^27^, ^29^

## LIMITATIONS

The study population consisted of a cohort of young, healthy volunteers from a relatively small sample size. This represents the greatest limitation to the generalizability and clinical application of this study, given this is not the typical patient population on which critical care resuscitation and intravascular volume assessment is performed. In addition, the EP sonographers acquiring data for the purposes of this study had training and experience beyond the average provider. Respiratory variation was measured during a rapid, forceful “sniff” as opposed to quiet respiration. IVC measures were performed in sequence (i.e. SA followed by LA, followed by CLA). Effect of diameter measured due to order of acquisition is unlikely; however, randomization of acquisition could have eliminated the potential for interaction or bias. Finally, collapsibility indices may be less useful clinically and evaluation of percentage of IVC collapse may prove more reliable. These conditions together may limit the generalizability of our findings, and further investigation and validation is warranted.

## CONCLUSION

POCUS of the IVC is a non-invasive means of volume assessment in the critically ill. Standardization and optimal techniques for IVC assessment have yet to be agreed upon. This study was designed to determine inter-rater reliability of ultrasound measurements between different views and modalities. These results suggest that B-mode LA holds the most promise to deliver reliable measures of IVC diameter. These data may help to establish a standardized approach to POCUS of the IVC for intravascular volume assessment. Future studies may focus on validation in a clinical setting as well as comparison to a reference standard.

## Figures and Tables

**Table 1:** Mean inferior vena cava (IVC) diameter by ultrasound view and mode.

View	Mode	Mean (SD)
View	B-mode	1.86 (0.42)
	IVCe	1.97 (0.47)
	IVCi	1.25 (0.45)
SA (cm)	B-mode	1.89 (0.43)
	IVCe	1.98 (0.46)
	IVCi	1.33 (0.49)
CLA (cm)	B-mode	1.98 (0.44)
	IVCe	2.02 (0.47)
	IVCi	1.41 (0.46)

*LA,* sub-xyphoid transabdominal long axis; *SA*, transabdominal short axis; *CLA,* right lateral transabdominal coronal long axis; *IVCe,* inferior vena cava expiration; *IVCi*, inferior vena cava inspiration

N = 117 ultrasound scans per mode.

**Table 2:** Interclass correlation coefficient by modality.

View	B-mode (95% CI)	IVCe (95% CI)	IVCi (95% CI)	IVCCI (95% CI)
LA	0.86 (0.76–0.92)	0.78 (0.60–0.88)	0.57 (0.19–0.78)	0.14 (−0.27–0.47)
SA	0.78 (0.63–0.88)	0.76 (0.53–0.88)	0.63 (0.28–0.81)	0.27 (−0.11–0.56)
CLA	0.74 (0.56–0.85)	0.68 (0.45–0.82)	0.66 (0.42–0.81)	0.32 (−0.08–0.60)

*LA,* sub-xyphoid transabdominal long axis; *SA*, transabdominal short axis; *CLA,* right lateral transabdominal coronal long axis; *IVCe,* inferior vena cava expiration; *IVCi*, inferior vena cava inspiration

N = 117 ultrasound scans per mode.
